# CO_2_-Responsive Worm-like Micelle Based on Double-Tailed Surfactant

**DOI:** 10.3390/ma18040902

**Published:** 2025-02-19

**Authors:** Fanghui Liu, Huiyu Huang, Mingmin Zhang, Meng Mu, Rui Chen, Xin Su

**Affiliations:** 1 Sinopec Key Laboratory of Drilling Completion and Fracturing of Shale Oil and Gas, Beijing 102206, China; liufh.sripe@sinopec.com; 2 CNOOC Institute of Chemicals & Advanced Materials (Beijing) Co., Ltd., Beijing 102209, China; 3 Zhejiang Research Institute of Tianjin University, Shaoxing 312369, China; 4 Shengli Oilfield Company, SINOPEC, Dongying 257092, China; 5 State Key Laboratory of Polymer Materials Engineering, Polymer Research Institute, Sichuan University, Chengdu 610065, China

**Keywords:** worm-like micelle, CO_2_-responsiveness, double-tailed surfactant, viscosity

## Abstract

CO_2_-responsive worm-like micelles (WLMs) are considered promising for applications in smart materials, enhanced oil recovery, and drug delivery because of their reversible and tunable properties. This study presents a novel system of CO_2_-responsive WLMs, which is constructed using a double-tailed surfactant (DTS). When exposed to CO_2_, the DTS molecules undergo protonation, resulting in the formation of ultra-long-chain cationic surfactants that self-assemble into worm-like micelles. The zero-shear viscosity of the DTS–CO_2_ solution achieves approximately 300,000 mPa·s, which is 300,000 times higher than that of pure water. In contrast, the DTS–air solution exhibits a viscosity of only 2 mPa·s. The system retains a viscosity above 100,000 mPa·s across a temperature range of 25–120 °C under a CO_2_ atmosphere. Moreover, it demonstrates reversible transitions between high- and low-viscosity states without any loss of responsiveness, even after multiple cycles. The critical overlap concentration of the DTS–CO_2_ micellar system is determined to be 80 mM. This research offers valuable insights into the development of CO_2_-responsive surfactants, highlighting their potential for designing advanced functional materials.

## 1. Introduction

Worm-like micelles [1,2] are flexible, long-chain nanostructures self-assembled by surfactant molecules in solution, named for their worm-like shape. They are typically formed by amphiphilic molecules with hydrophilic and hydrophobic segments through spontaneous hydrophobic interactions and electrostatic forces. The length of these micelles can reach the micrometer scale, while their diameter is usually in the nanometer range, exhibiting high flexibility and dynamic behavior. Worm-like micelles demonstrate unique rheological properties in solution, such as viscoelasticity and shear-thinning effects, making them widely applicable in fields such as cosmetics, drug delivery [3,4], oil recovery [5,6,7], and friction reduction [8,9,10].

Stimuli-responsive worm-like micelles with tunable rheological properties have attracted increasing attention. Common methods for forming worm-like micelles via external stimuli include pH [11,12], temperature [9,13], photochemistry [14,15], redox reactions [16], and the most popular, CO_2_ [17]. Upon exposure to these stimuli, the solution viscosity rapidly increases to its maximum value. Subsequently, by introducing another stimulus or removing the initial stimulus, the high-viscosity system can return to its original low-viscosity state. Due to this unique “on–off” switchable and recyclable behavior, stimuli-responsive worm-like micelles have shown great potential for applications.

CO_2_-responsive materials have emerged in recent years as a green innovation in chemical research, originating from chemists’ exploration of CO_2_ absorbents for greenhouse gas mitigation. These materials are a novel class of substances whose molecular structures can be altered by introducing CO_2_ gas, thereby controlling the surface properties of their solutions [18,19]. As a greenhouse gas, CO_2_ is abundant, relatively inexpensive, and easy to handle. Additionally, CO_2_ dissolves in water and can be easily removed without accumulation, posing no environmental pollution risks [20]. CO_2_-responsive worm-like micelles are essentially formed when neutral substances in aqueous solutions interact with CO_2_ and become protonated, imparting cationic surfactant-like properties to the neutral molecules. This triggers aggregation behavior in the solution, leading to the transition from spherical micelles to worm-like micelles, which macroscopically manifests as a significant increase in solution viscosity.

**Scheme 1:**
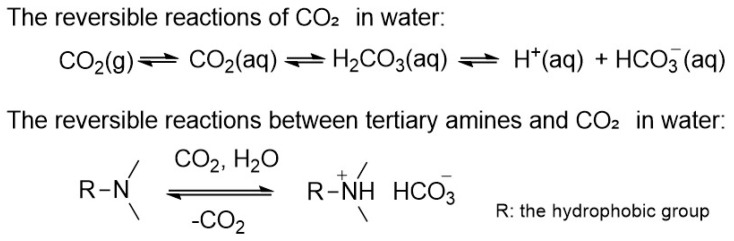
Reversible reaction of tertiary amines with CO_2_ in water.

The CO_2_-responsive mechanism is as follows: when CO_2_ gas is introduced into an aqueous solution of tertiary amines, CO_2_ exists in both free and dissolved states, reaching a gas-liquid equilibrium (Scheme 1). The basic tertiary amines react with dissolved CO_2_ in the solution through an acid-base neutralization reaction, forming bicarbonate structures, while the tertiary amine groups are protonated into quaternary ammonium cation structures. This enhances the hydrophilicity of the amine groups. We can introduce another stimulus into the solution in order to decrease the free CO_2_ concentration., disrupting the gas-liquid equilibrium. As the equilibrium shifts to the left (Scheme 1), the bicarbonate structure decomposes, and the quaternary ammonium structure undergoes deprotonation to revert to tertiary amine groups. Jessop group [18] first reported a CO_2_-switchable surfactant containing amidine groups. Before introducing CO_2_ gas, the material exhibited low hydrophilicity and lacked surfactant properties. However, upon CO_2_ introduction, highly hydrophilic amidinium bicarbonate ionic head groups were formed, which, combined with the hydrophobic alkyl chains, constituted a surfactant structure capable of significantly reducing the surface tension of aqueous solutions and exhibiting surfactant properties. Subsequently, this switchable surfactant was applied to emulsify and reduce the viscosity of heavy oil, becoming a research hotspot both domestically and internationally. Zhang et al. [21] prepared a viscoelastic “pseudo-gemini” surfactant under CO_2_ stimulation using a mixed solution of N,N,N′,N′-tetramethyl-1,3-propane diamine (TMPDA) and sodium dodecyl sulfate (SDS) at a molar ratio of 1:2. When CO_2_ was introduced into the mixed solution, the tertiary amine TMPDA was protonated, resulting in positively charged groups at both ends of the molecule. These molecules then self-assembled with SDS in solution through electrostatic attraction to form worm-like micelles, exhibiting excellent viscoelasticity. When N_2_ was introduced, or the solution was heated to remove CO_2_, the positively charged TMPDA underwent deprotonation to revert to neutral tertiary amines, separating from SDS. The worm-like micelles transitioned back into spherical micelles, and the system returned to its original low-viscosity state. Su et al. [22] prepared a CO_2_-responsive anionic worm-like micelle system using sodium octadecyl sulfate (C_18_H_37_SO_4_Na) and N,N-dimethylethanolamine (DMAE) at 60 °C. Under CO_2_ stimulation, DMAE was protonated to carry a positive charge, and it self-assembled with the anionic surfactant C_18_H_37_SO_4_Na through electrostatic attraction to form worm-like micelles. When N_2_ was introduced, the high-viscosity system reverted to its initial low-viscosity state. This process could be repeated more than three times, with minimal variation in the maximum viscosity achieved. By simply mixing two commercial reagents (such as stearic acid and cyclen), the formation of CO_2_-switchable pseudo-tetrameric surfactants was successfully achieved, which subsequently self-assembled into CO_2_-switchable worm-like micelles. Through the use of CO_2_, the rheological properties were cycled between a low-viscosity fluid and a viscoelastic fluid [23]. Most of the existing CO_2_-responsive surfactants possess conventional surfactant structures, with only a single tail chain in their molecular structure.

Double-tail surfactants are typically composed of two hydrophobic tail chains and a single hydrophilic head group. Compared to conventional single-tail surfactants, double-tail surfactants contain two alkyl chains, resulting in a larger hydrophobic moiety and stronger intermolecular hydrophobic interactions. Since hydrophobic interactions are the primary driving force for surfactant self-assembly [24], the two hydrophobic chains of double-tail surfactants facilitate the self-assembly process. According to the packing parameter theory, such surfactants tend to form vesicles and lamellar structures [25,26]. Due to the similarity of these aggregates to phospholipid bilayers, double-tail surfactants are often used to mimic biological membranes for studying processes such as transmembrane transport. In addition, compared to single-tail surfactants, double-tail surfactants generally exhibit higher interfacial activity, with enhanced dispersion, emulsification [27], and wetting [28] capabilities. As a result, double-tail surfactants hold great potential for applications in fields such as detergents [29] and drug delivery [30]. However, the larger hydrophobic moiety also contributes to an increased Krafft point and reduced water solubility, which significantly limits their applications.

Based on the aforementioned characteristics of double-tail surfactants, we aim to design and synthesize CO_2_-responsive double-tail surfactants and investigate their ability to form worm-like micelles. By incorporating CO_2_-responsive functional groups into the molecular structure, these surfactants can exhibit tunable self-assembly behavior under external CO_2_ stimuli. The presence of two hydrophobic tail chains is expected to enhance the hydrophobic interactions, facilitating the formation of worm-like micelles and potentially improving the structural stability and responsiveness of aqueous solutions.

## 2. Experimental Procedures

### 2.1. Materials

Diethyl malonate, 1-bromododecane, sodium hydride, 3-dimethylaminopropylamine, and thionyl chloride were purchased from Sigma-Aldrich (St. Louis, MI, USA). Carbon dioxide (CO_2_, purity ≥ 99.998%) and nitrogen (N_2_, purity 99.998%) were used without further purification. Throughout the study, deionized water with a resistivity of 18.25 MΩ·cm was provided by a CDUPT-III ultrapure water purifier (Chengdu Ultrapure Technology Co., Ltd., Chengdu, China).

### 2.2. Preparation of Surfactant Solution

An appropriate amount of DTS was accurately weighed and dissolved in a specified volume of triple-distilled water. The mixture was heated in a water bath until the waxy solid melted and formed a liquid oil film. CO_2_ gas at a pressure of 0.1 MPa and a flow rate of approximately 0.1 L·min^−1^ was introduced into the system at room temperature. The mixture was continuously shaken until a homogeneous and transparent solution was obtained. The system was sealed and stored at room temperature, and the resulting solution was labeled as “DTS–CO_2_”. A series of diluted solutions were treated with CO_2_ gas for 10–15 s with continuous shaking. The resulting solutions were then sealed and stored.

The preparation of DTS–air involved introducing air at a pressure of 0.1 MPa and a flow rate of approximately 0.1 L·min^−1^ into 10 mL of the DTS–CO_2_ solution for approximately 20 min. This process resulted in the formation of a low-viscosity emulsion.

### 2.3. pH Measurement

The pH of the sample was measured using the S2-T Kit pH meter (Mettler Toledo, Zurich, Switzerland). Prior to the measurement, the electrode of the pH meter was thoroughly rinsed with deionized water. During the measurement, the pH meter reading was recorded at 25 °C after stabilization. The measurement was repeated three times, and the average value was calculated.

### 2.4. Conductivity Measurement

The conductivity of aqueous solutions was measured using an EF30 conductometer (Mettler Toledo, Mississauga, ON, Canada) at 25 °C. The reagent bottle containing the sample was placed in a thermostatic water bath maintained at the specified temperature. After 30 min, the conductivity measurement electrode was immersed into the sample solution. The solution was allowed to reach the set temperature and remain stable before further measurements were conducted. The measurement button was pressed to initiate the conductivity measurement, and the corresponding conductivity value was recorded. Each sample was measured in triplicate, ensuring a relative deviation of less than 2%. The average value of the three replicate measurements was calculated and reported as the conductivity value of the corresponding sample.

### 2.5. Rheological Property Testing

The rheological properties of the samples were analyzed using a Physica MCR301 rheometer (Anton Paar, Graz, Austria) equipped with a CC27 concentric cylinder rotor (radius: 13.33 mm) and a stator (radius: 14.46 mm). The temperature of the sample was controlled using a Peltier system with an accuracy of ±0.01 °C, while a solvent trap was used to minimize evaporation. The instrument was calibrated using Cannon standard oil prior to testing. The samples were equilibrated in a water bath at the specified temperature for 1 h and then stabilized in the stator for 5 min prior to testing. Steady shear experiments were conducted at shear rates ranging from 10^−3^ to 10^2^ s^−1^. Dynamic rheological tests included stress sweeps performed at 1 Hz to identify the linear viscoelastic region, followed by frequency sweeps ranging from 0.05 to 100 rad·s^−1^.

### 2.6. Cryo-TEM Observation

Firstly, the surfactant solution was prepared by dissolving it to an appropriate concentration and adding a cryoprotectant to minimize ice crystal formation. The solution was then applied onto a cryo-carrier to form a thin layer, followed by rapid freezing to liquid nitrogen temperatures using techniques such as high-pressure freezing or plunge freezing. Finally, the frozen sample was transferred to a cryo-transfer device, such as a cryo-transfer rod, for observation under low-temperature conditions in the electron microscope.

The microstructure of the micelle solutions was examined using a cryogenic transmission electron microscope (Cryo-TEM, model JEM2010, JEOL Ltd., Tokyo, Japan). The operating conditions of the instrument included an accelerating voltage of 200 kV, a CCD imaging system for image acquisition with a Gatan 832 camera (Gatan, Inc., Pleasanton, CA, USA), and a cryo-transfer holder (Gatan-626, Gatan, Inc., Pleasanton, CA, USA) that maintained a temperature not exceeding −174 °C. The microgrid employed for sample preparation was Quantifoil 1.2/1.3 (Quantifoil Micro Tools GmbH, Jena, Germany).

## 3. Results

**Scheme 2:**
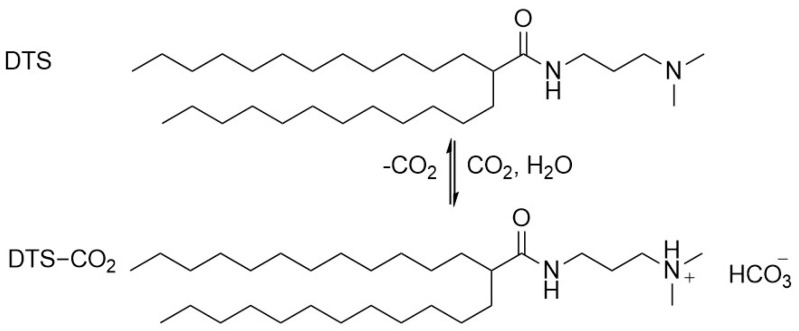
Reversible reaction of DTS with CO_2_.

A double-tail CO_2_-responsive surfactant, referred to as DTS, was synthesized (Scheme S1) to study its ability to form worm-like micelles. The incorporation of CO_2_-responsive functional groups into the molecular structure enables this surfactant to exhibit adjustable self-assembly behavior when exposed to CO_2_ stimulation, which is named DTS-CO_2_ (Scheme 2). The presence of double hydrophobic tail chains enhances intermolecular hydrophobic interactions, which facilitates the formation of worm-like micelles and potentially improves both the stability and responsiveness of the system. This study aims to investigate the self-assembly behavior of this novel surfactant under CO_2_ stimulation, its capacity to form worm-like micelles, and its rheological properties.

### 3.1. CO_2_-Responsive Properties of DTS

**Figure 1:**
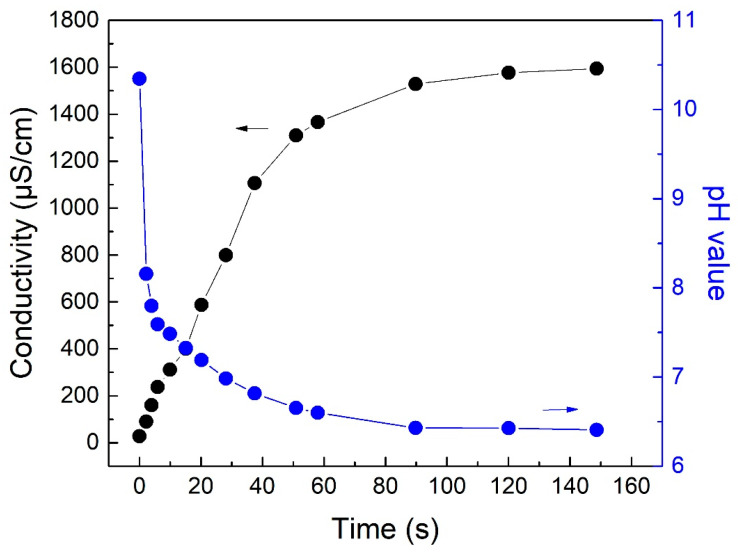
Conductivity and pH values of 100 mM DTS solution change with increasing bubbling CO_2_ time.

To determine whether the DTS with terminal amine group exhibits CO_2_-responsive characteristics comparable to those of small-molecule primary amines, the variations in macroscopic conductivity and pH of the DTS solution during CO_2_ bubbling were initially analyzed. The results are presented in Figure 1. When CO_2_ gas was continuously bubbled into 20 mL of a 100 mM DTS aqueous solution at a constant rate of 0.1 L·min^−1^, the pH value decreased sharply at first, then gradually slowed and eventually stabilized at 6.42, reaching equilibrium. At the same time, the conductivity increased sharply from 3.2 μS·cm^−1^ and ultimately stabilized at 1586 μS·cm^−1^. This observation indicates that when CO_2_ reacts with water, it interacts with the primary amine groups in the DTS molecular structure, producing a significant number of ionized compounds. This reaction neutralizes the alkalinity of the solution, leading to a decrease in pH and an increase in conductivity. These preliminary experimental results confirm that DTS exhibits CO_2_-responsive characteristics. Based on the experimental data from conductivity and pH measurements, it can be concluded that DTS molecules in an aqueous solution undergo the reversible reaction illustrated in Scheme 1.

**Figure 2:**
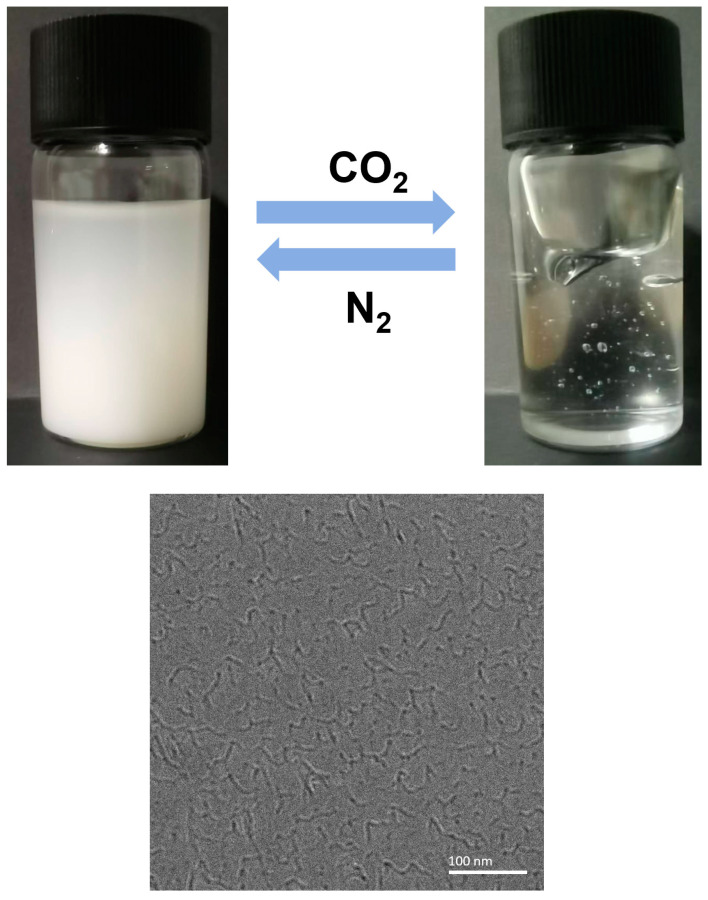
(**Upper**) Appearance of 100 mM DTS solution after bubbling CO_2_ and removing CO_2_ with N_2_; (**Lower**) The Cryo-TEM image of worm-like micelles based on 100 mM DTS with CO_2_ at 25 °C.

As DTS undergoes protonation by CO_2_, transitioning from a nonionic state to an ionic state, its hydrophilicity is significantly enhanced. The ionic headgroup and hydrophobic tail of the resulting molecule form a typical ultra-long-chain cationic surfactant. Surfactants containing double-tails have been shown to exhibit a strong ability to form worm-like micelles, making them highly effective rheological thickening agents [31]. Figure S1 shows that the critical micellular concentration (CMC) of DTS-CO_2_ is approximately 0.009 mM. Consequently, a 100 mM DTS solution is anticipated to form a worm-like micellar system exhibiting CO_2_-responsive characteristics. Figure 2 depicts the changes in the physical appearance of a 100 mM DTS solution during the introduction and subsequent removal of CO_2_. Before the introduction of CO_2_, DTS exhibits extremely poor water solubility. At low temperatures, it appears as a waxy solid suspended on the water surface, while at higher temperatures, it forms an oil film on the water surface. Upon bubbling CO_2_ gas into 20 mL of a 100 mM DTS solution at a rate of approximately 0.1 L·min^−1^ at room temperature for 2 min, the solution rapidly transitions from a biphasic system to a transparent and homogeneous viscoelastic fluid (DTS–CO_2_), capable of trapping bubbles for an extended duration. When N_2_ is introduced into the DTS–CO_2_ solution to displace CO_2_, or when the solution is exposed to air for 7 days, the viscous fluid rapidly transforms into a low-viscosity emulsion. This process is reversible and can be repeated multiple times without any loss of responsiveness.

**Figure 3:**
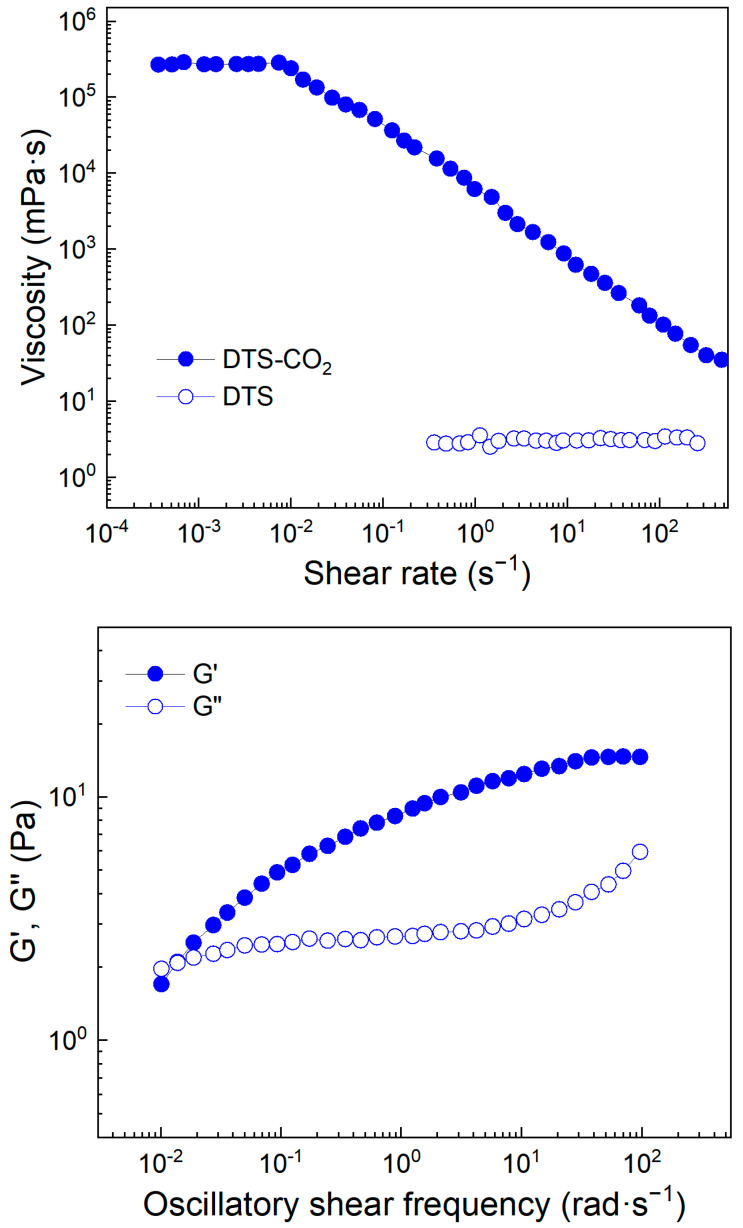
Steady and dynamic rheology of 100 mM DTS-CO_2_ solution at 25 °C.

Considering these visually significant changes, rheological methods were employed to further evaluate the CO_2_-responsive behavior. As illustrated in Figure 3, the steady-state rheological behavior of the DTS–CO_2_ solution displays Newtonian fluid characteristics at shear rates below 0.01 s^−1^, with an apparent viscosity reaching approximately 300,000 mPa·s, which is about 300,000 times that of pure water. When the shear rate exceeds 0.01 s^−1^, the solution exhibits distinct shear-thinning behavior. Furthermore, dynamic rheological results reveal that when the oscillatory shear frequency (ω) is below approximately 0.018 rad·s^−1^, the elastic modulus (G′) is smaller than the viscous modulus (G″), suggesting that the solution behaves predominantly as a viscous fluid. When the oscillatory shear frequency exceeds approximately 0.018 rad·s^−1^, G′ surpasses G″, and the solution transitions to exhibit elastic behavior. In summary, the DTS–CO_2_ solution exhibits pronounced viscoelastic responsiveness. Both steady-state and dynamic rheological analyses indicate that the DTS–CO_2_ solution contains a three-dimensional network structure formed by entangled worm-like micelles at the microscopic level. In contrast, both the DTS–air solution and the pure DTS solution exhibit typical Newtonian fluid behavior, with zero-shear viscosities (η_0_) of approximately 2 mPa·s, respectively, which are merely one hundred-thousandth of that of the DTS–CO_2_ solution. Moreover, the G′ and G″ values of these solutions are exceedingly low, rendering them challenging to measure via dynamic rheological experiments.

**Figure 4:**
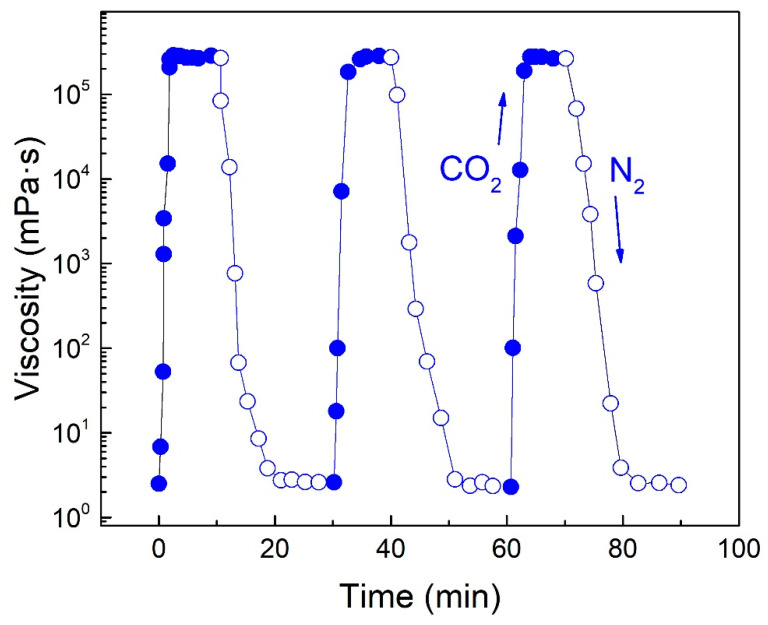
Zero-shear viscosity (η_0_) of the 100 mM DTS solution measured during the repeated cycles of bubbling CO_2_ and N_2_ at 25 °C. The solid data points represent the conductivity when CO_2_ is introduced, while the hollow data points represent the conductivity when N_2_ is introduced.

More importantly, the stimulus-responsive behavior of the DTS solution to CO_2_ and air is fully reversible, enabling its viscosity to be switched in a controllable manner by bubbling CO_2_ or air. As shown in Figure 4, after multiple cycles of states with or without CO_2_, the η_0_ of the solution can be fully restored to its original “on” value of 300,000 mPa·s and “off” value of 2 mPa·s. Compared to previous CO_2_-switchable systems, this reversible cycling process is easily achieved by bubbling CO_2_ or air at room temperature, eliminating the need for deliberate heating or the use of inert gases such as nitrogen or argon. This system is economically viable, environmentally friendly, and energy-efficient.

To gain a deeper understanding of the microscopic micellar structures within the solution, Cryo-TEM was utilized to examine the DTS–CO_2_ solution. As depicted in Figure 2, high-density worm-like micelles are distinctly observed in the DTS–CO_2_ solution. These micelles exhibit diameters of several nanometers and lengths extending to hundreds of nanometers. The three-dimensional network structure, formed by the entanglement of these worm-like micelles, is responsible for the solution’s exceptional rheological viscoelasticity. In summary, a CO_2_-air switchable worm-like micellar system has been successfully constructed based on DTS.

### 3.2. Factors Affecting the Rheological Properties of Worm-like Micelles

To comprehensively examine the influence of environmental factors and various conditions on the rheological properties of the solution, a series of experiments were performed. Initially, an experiment focusing on CO_2_ bubbling time was conducted to investigate the dynamic evolution of solution viscosity over time during CO_2_ introduction. The results demonstrated that the viscosity increased rapidly with the degree of protonation until it eventually reached a stable state. Furthermore, additional experiments examined the viscoelastic stability of the solution under varying conditions, such as changes in concentration, temperature, and pressure.

#### 3.2.1. CO_2_ Sparging Time

**Figure 5:**
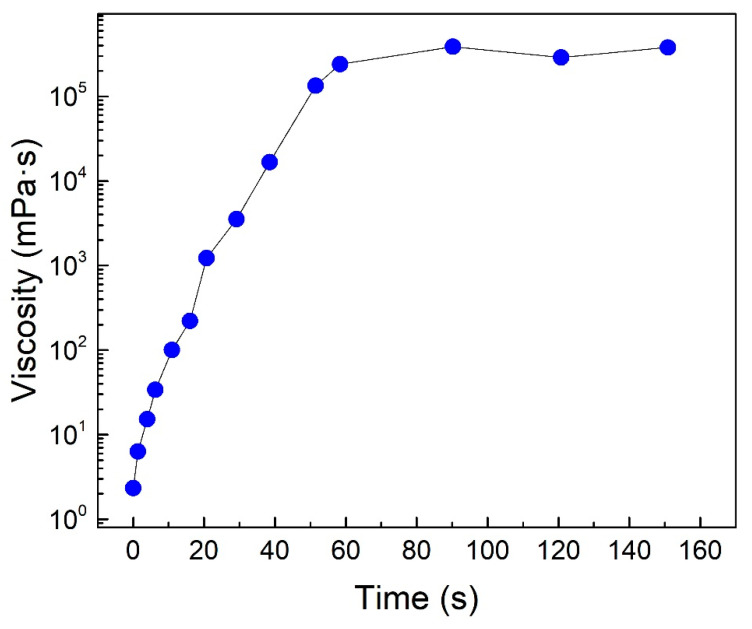
Zero-shear viscosity of 20 mL of a 100 mM DTS solution changes with the time of sparging CO_2_ at a flow rate of 0.1 L/min.

Figure 5 illustrates the relationship between zero-shear viscosity and time for a 100 mM aqueous solution of DTS (20 mL), with CO_2_ gas sparged in at a constant rate of approximately 0.1 L·min^−1^ for varying durations. The viscosity of the solution increases exponentially as the CO_2_ bubbling time extends. After around 60 s, the viscosity reaches its peak value, and the system attains equilibrium. This suggests that within the initial 0–60 s, CO_2_ bubbling induces the protonation of DTS molecules, converting them into long-chain cationic surfactants. As the bubbling time increases, the concentration of cationic surfactants in the system rises correspondingly. The viscosity of worm-like micelle solutions generally follows an exponential relationship with the concentration of surfactants. When the CO_2_ bubbling time reaches 60 s, nearly all DTS molecules in the system have completely reacted, and the concentration of surfactants remains constant. As a result, the viscosity of the solution stabilizes, mirroring the trends observed for pH and conductivity (Figure 1). The viscosity of a 100 mM DTS solution can reach 10^5^ mPa·s, whereas conventional surfactant solutions at a concentration of 300 mM only achieve a viscosity of 10^3^ mPa·s [23]. This clearly highlights the advantage of the double-tail structure utilized in the molecular design of DTS.

#### 3.2.2. Concentration of DTS

**Figure 6:**
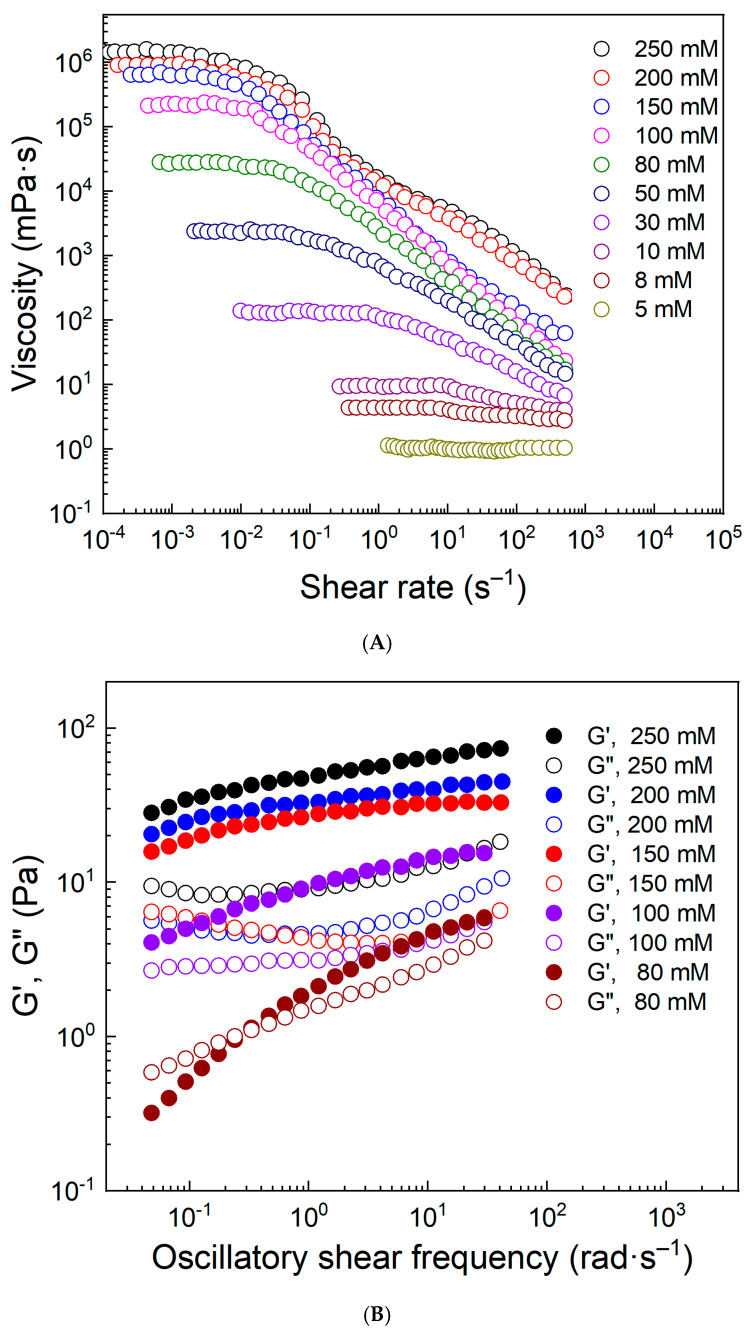
Steady (**A**) and dynamic (**B**) rheology of DTS–CO_2_ solutions with different concentrations at 25 °C.

Figure 6 illustrates the rheological properties of DTS aqueous solutions at various concentrations after CO_2_ was introduced to saturation under room temperature conditions. The steady-state rheological curves in Figure 6A indicate that at a concentration of 5 mM, the solution exhibits typical Newtonian fluid behavior. At a concentration of 8 mM, the solution begins to exhibit shear-thinning behavior. With increasing concentration, the Newtonian viscosity plateau rises gradually while the critical shear rate for the onset of shear thinning decreases. This suggests that an entangled network structure forms within the system, with higher surfactant concentrations resulting in denser networks. These denser networks are more susceptible to shear strain, leading to the realignment of worm-like micelles along the direction of the shear stress field. The dynamic rheology results (Figure 6B) demonstrate that all samples exhibit pronounced viscoelasticity. At a concentration of 80 mM, the G′ and G″ intersect at approximately 0.3 rad·s^−1^. At a concentration of 100 mM, G′ and G″ do not intersect within the measured frequency range, and G′ consistently remains higher than G″, indicating gel-like rheological behavior.

**Figure 7:**
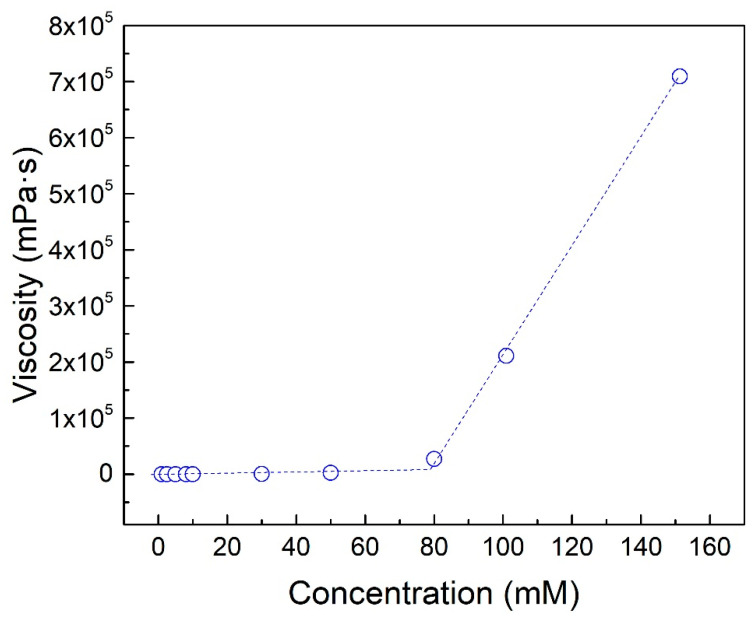
Effect of DTS–CO_2_ concentrations on zero–shear viscosity (η_0_) at 25 °C.

Figure 6 provides the basis for deriving the rheological parameters of DTS–CO_2_ solutions as a function of surfactant concentration, which are presented in Figure 7. The variation of η_0_ with concentration. The η_0_–C curve can be distinctly divided into two regions by a turning point. The concentration at this turning point represents the critical overlap concentration (C∗) of the DTS–CO_2_ worm-like micellar system, showing a value of 80 mM. In the dilute solution region (C < C∗), η_0_ increases linearly with concentration, following Einstein’s viscosity equation. In the semi-dilute solution region (C > C∗), η_0_ exhibits a proportional relationship with concentration, following η_0_~C. The increase in these rheological parameters is primarily attributed to the elongation of worm-like micelles. With increasing surfactant concentration, the length and flexibility of worm-like micelles gradually increase, leading to a denser network structure.

#### 3.2.3. Pressure and Temperature

**Figure 8:**
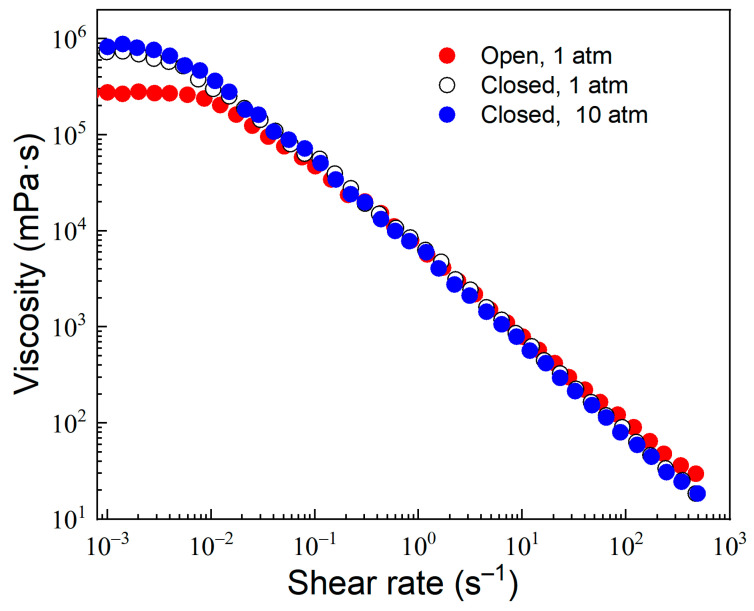
Steady rheology of 100 mM DTS–CO_2_ solution under different pressures and situations.

The DTS–CO_2_ solution exhibits high viscosity under a CO_2_ atmosphere, with its potential application being the sealing and plugging of CO_2_ in geological formations. However, the pressure in geological formations is typically very high. Therefore, it is necessary to evaluate the thickening behavior of the DTS solution under high CO_2_ pressure, such as 10 atm CO_2_ atmosphere. The rheological behavior of the DTS–CO_2_ solution was analyzed in a sealed CO_2_ atmosphere using a high-pressure closed rheological system. Figure 8 illustrates the steady-state rheological curves of the DTS–CO_2_ solution under varying pressures within a sealed CO_2_ environment. A comparison with the curves obtained in an open environment reveals that the η_0_ of the system increases slightly in the sealed environment, though the increase is not statistically significant. Moreover, increasing the environmental pressure does not result in any further changes in η_0_. This finding suggests that the DTS–CO_2_ solution demonstrates greater stability in a CO_2_ atmosphere while remaining unaffected by pressure, further confirming its sensitivity to air exposure.

**Figure 9:**
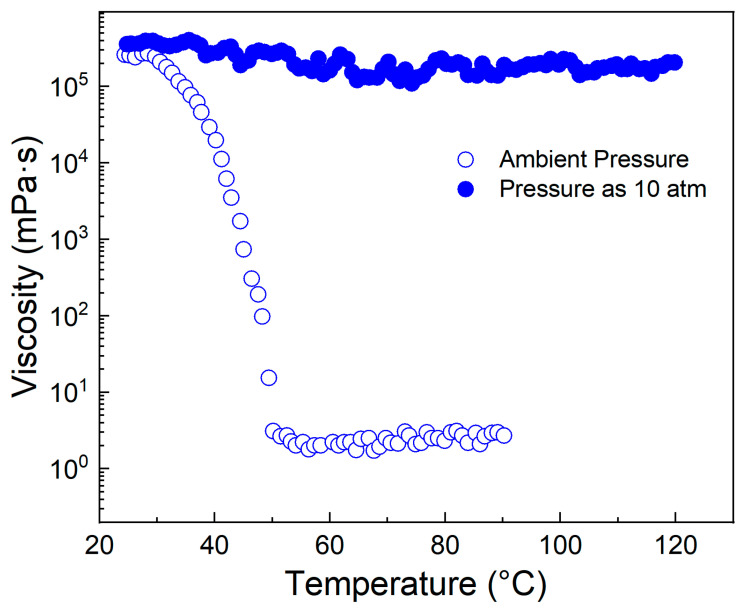
Zero-shear viscosity of 100 mM DTS–CO_2_ solution changes with increasing temperature at a fixed shear rate of 0.005 s^−1^.

Figure 9 illustrates the variation in the apparent viscosity of the DTS–CO_2_ solution as a function of temperature. In an open environment, the viscosity decreases rapidly as the temperature increases. At approximately 55 °C, the viscosity reaches its minimum value, which is comparable to that of pure water. Further increases in temperature result in negligible changes in viscosity. In contrast, under a 10 atm CO_2_ atmosphere, the solution’s viscosity exhibits slight fluctuations with increasing temperature but remains above 100,000 mPa·s across the temperature range of 25–120 °C. This comparison highlights the excellent thermal stability of the DTS–CO_2_ solution in a CO_2_ atmosphere.

## 4. Conclusions

This study reports the design and synthesis of a CO_2_-responsive double-tailed surfactant, DTS, and examines its capacity to form worm-like micelles under CO_2_ stimulation, along with its rheological properties. The findings indicate that DTS molecules undergo protonation when exposed to CO_2_, which significantly enhances their hydrophilicity and leads to the formation of ultra-long-chain cationic surfactants. These surfactants self-assemble into worm-like micelles and exhibit notable rheological viscoelasticity. Specifically, the zero-shear viscosity of the DTS–CO_2_ solution is approximately 300,000 mPa·s, which is 300,000 times higher than that of pure water. In contrast, the DTS–air solution and pure DTS solution exhibit η_0_ values of only 2 mPa·s. The system exhibits excellent thermal stability, maintaining a viscosity above 100,000 mPa·s within a temperature range of 25–120 °C under a CO_2_ atmosphere. It also shows high sensitivity to CO_2_ concentration, surfactant concentration, and bubbling time. Moreover, the system allows a reversible transition between high-viscosity and low-viscosity states through the bubbling of CO_2_ or air, with no observable loss of responsiveness after multiple cycles. The critical overlap concentration of the DTS–CO_2_ worm-like micellar solution is found to be 80 mM. At higher concentrations, the solution exhibits shear-thinning behavior and pronounced viscoelasticity.

Compared to existing CO_2_-responsive systems, the DTS–CO_2_ system offers simplicity, environmental friendliness, and cost-effectiveness, as it does not require heating or inert gases for reversibility. These characteristics make it a promising candidate for applications in smart materials, enhanced oil recovery, and other areas where tunable rheological properties are essential. The study offers valuable insights into the development of advanced functional materials that leverage CO_2_ responsiveness.

## Data Availability

The raw data supporting the conclusions of this article will be made available by the authors on request.

## Supplementary Materials

The following supporting information can be downloaded at https://www.mdpi.com/article/10.3390/ma18040902/s1, Scheme S1. Preparation Steps for CO_2_-Responsive DTS Surfactants; Figure S1. The I_1_/I_3_ values obtained by fluorescence spectroscopy as a function of DTS–CO_2_ concentration at 25 °C.
