# Supplementary material for: CO2-Responsive Worm-like Micelle Based on Double-Tailed Surfactant
Source: Materials (Basel). 2025 Feb 19;18(4):902. doi: 10.3390/ma18040902 (PMC11857229; doi:10.3390/ma18040902)
Supplement: Supplementary file 1 [file materials-18-00902-s001.zip › materials-3482790-supplementary.pdf]

Supporting Information for

CO_2-Responsive Worm-like Micelle Based on Double-Tailed Surfactant

Fanghui Liu [1], Huiyu Huang [2], Mingmin Zhang [3,*], Meng Mu [4,*], Rui Chen [3] and Xin Su [5]

[1] Sinopec Key Laboratory of Drilling Completion and Fracturing of Shale Oil and Gas, Beijing 102206, China
[2] CNOOC Institute of Chemicals & Advanced Materials (Beijing) Co., Ltd., Beijing 102209, China
[3] Zhejiang Research Institute of Tianjin University, Shaoxing 312369, China
[4] Shengli Oilfield Company, SINOPEC, Dongying 257092, China
[5] State Key Laboratory of Polymer Materials Engineering, Polymer Research Institute, Sichuan University, Chengdu 610065, China
* Correspondence: mingmin_zhang2000@163.com (M.Z.); mumeng_cas@163.com (M.M.)

The detailed synthetic process is described as follows [1,2], shown in Scheme S1.

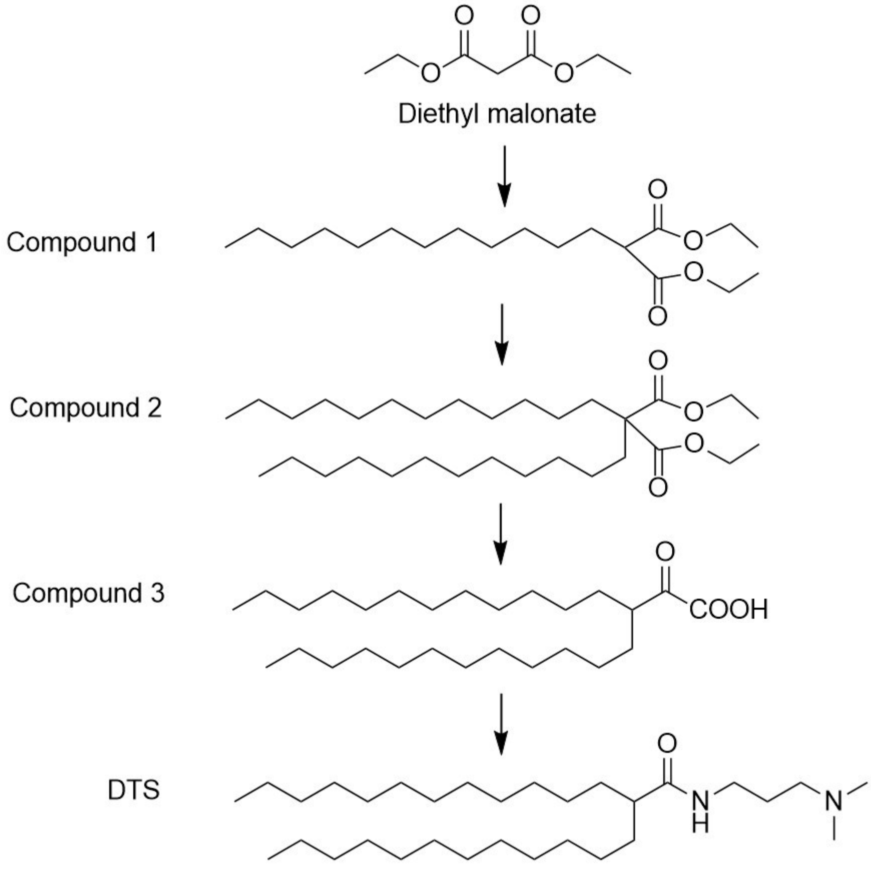

Scheme S1. Preparation Steps for CO_2-Responsive DTS Surfactants

(1) Synthesis of Compound 1.

The three-necked flask was charged with 60 wt.% sodium hydride (31.3 g, 0.78 mol) and a small amount of petroleum ether. After the precipitation of sodium hydride, the supernatant was removed, and 500 mL of tetrahydrofuran (THF) was introduced. Diethyl malonate (125.3 g, 0.78 mol) was gradually introduced into the system at room temperature under continuous stirring, leading to the evolution of hydrogen gas. Upon completion of the addition, the reaction mixture was stirred for an additional 2 hours. Subsequently, 1-bromododecane (150 g, 0.60 mol) was added, and the reaction was maintained at 70°C for 6 hours. After the reaction mixture was cooled to room temperature, an aqueous solution of citric acid was introduced. Following phase separation, the organic layer was washed multiple times with ultrapure water until neutral pH (pH 7) was achieved, and subsequently dried over anhydrous magnesium sulfate ($MgSO_4$). Inorganic salts and the solvent were removed, and the resulting residue was purified by vacuum distillation to yield a light-yellow viscous liquid, identified as Compound 1. The yield was 70%.

(2) Synthesis of Compound 2.

A three-necked flask was charged with 60 wt.% sodium hydride (17 g, 0.43 mol) and a small amount of petroleum ether. After the precipitation of sodium hydride, the supernatant was removed, and 600 mL of tetrahydrofuran (THF) was introduced. Compound 1 (105.8 g, 0.32 mol) was gradually added dropwise at 60°C, resulting in the evolution of hydrogen gas. Upon completion of the addition, the reaction mixture was stirred for an additional 3 hours. Subsequently, 1-bromododecane (84.3 g, 0.34 mol) was introduced, and the reaction was maintained at 72°C for 6 hours. After cooling the reaction mixture to room temperature, an aqueous solution of citric acid was introduced. Following phase separation, the organic layer was washed multiple times with ultrapure water until neutral pH (pH 7) was achieved, and subsequently dried over anhydrous magnesium sulfate ($MgSO_4$). Inorganic salts and the solvent were removed, and the resulting residue was purified by vacuum distillation to yield a light-yellow viscous liquid, identified as Compound 2. The yield was 51%.

(3) Synthesis of Compound 3.

A three-necked flask was charged with sodium hydroxide (23.3 g, 0.58 mol) and 300 mL of ethanol, which was heated to 70°C to dissolve the base. Compound 2 (72.3 g, 0.15 mol) was subsequently introduced, and the reaction mixture was stirred at 80°C for 24 hours. If the system became too viscous, a water/ethanol mixed solvent was added. After the reaction, the solvent was removed, and 150 mL of deionized water was introduced. The pH of the mixture was adjusted to 2 using concentrated hydrochloric acid, and the mixture was stirred at 50°C for 2 hours. After filtration, the solid obtained was heated to 180°C in a single-necked flask, during which a large amount of gas was released. After 4 hours, the reaction was completed. The crude product was recrystallized three times with methanol and vacuum-dried to yield a light yellow

powdery solid, identified as Compound 3. The yield was 55%.

(4) Synthesis of DTS

Compound 3 (33.7 g, 0.08 mol) was charged into a three-necked flask, followed by the addition of a few drops of DMF. Thionyl chloride (12.1 g, 0.10 mol) was subsequently added dropwise at 60°C. The acidic gas generated during the reaction was absorbed using an aqueous sodium hydroxide solution. The reaction mixture gradually became a clear liquid. Excess thionyl chloride (SOCl2) was removed under reduced pressure, affording yellow viscous liquid 1. A three-necked flask was charged with an appropriate amount of dichloromethane, triethylamine (73.8 g, 0.73 mol), and 3-(dimethylamino)-1-propylamine (27.3 g, 0.26 mol), and the mixture was stirred in an ice bath. The yellow viscous liquid 1 (114.5 g, 0.24 mol) was gradually introduced into the system dropwise. Upon completion of the addition, the reaction mixture was stirred at room temperature for 6 hours. Following the reaction, an appropriate amount of dichloromethane and deionized water was used for extraction. The crude product was purified by recrystallization (three times) using ethanol and subsequently dried under vacuum at 55°C to afford DTS as a white solid. The yield was 71%.

In the presence of CO_2 and water, DTS undergoes protonation to form the cationic surfactant DTS-CO_2, which, similar to other surfactants, has a critical micelle concentration (CMC). As illustrated in Figure S1, the CMC of the cationic surfactant DTS-CO_2 was determined using pyrene as a fluorescence probe. As the concentration of DTS-CO_2 increased, the I_1/I_3 ratio initially decreased gradually and then exhibited a sharp decline upon reaching the critical concentration. The concentration at the inflection point represents the CMC of the surfactant, which is approximately 0.009 mM. This result indicates that DTS-CO_2 molecules, similar to other ultra-long-chain surfactants, begin to self-assemble at very low concentrations.

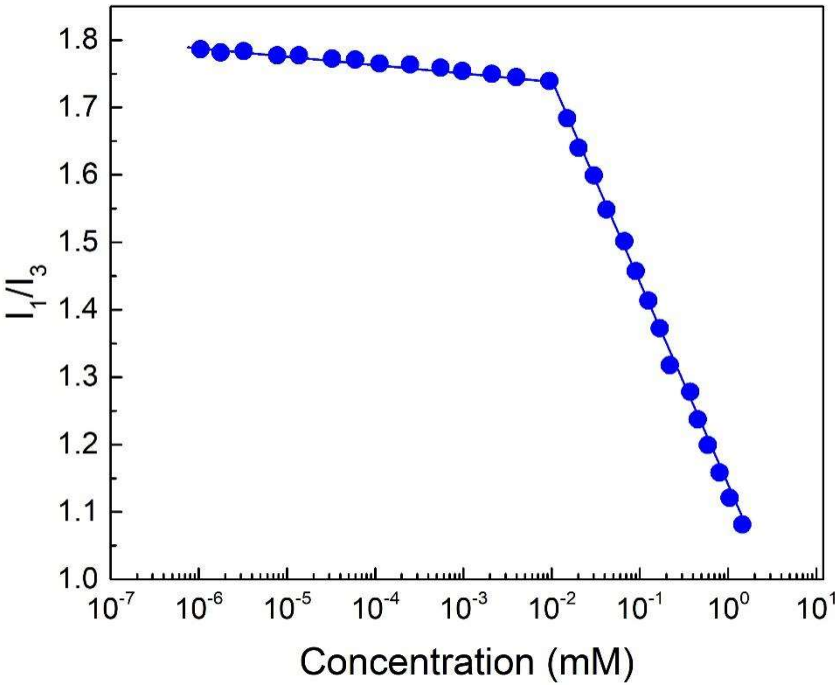

Figure S1. The I_1/I_3 values obtained by fluorescence spectroscopy as a function of DTS–CO_2 concentration at 25 °C.

Reference:

1. Lin, Z.; Bi, Z.; Li, H.; Pei, X.; Chen, Z.; Cui, Z.; Song, B. Wormlike micellar glycerol solutions formed from a double-tailed surfactant with two quaternary ammonium head groups. *Langmuir* **2024**, *40(38)*, 19954-19963.
2. Li, H.; Lin, Z.; Chen, Z.; Cui, Z.; Song, B. Wormlike micellar solutions formed by an anionic surfactant and a cationic surfactant with two head groups. *Soft Matter,* **2024**, *20*, 978-984.
